# Elevated blood lead levels among unskilled construction workers in Jimma, Ethiopia

**DOI:** 10.1186/1745-6673-9-12

**Published:** 2014-03-19

**Authors:** Higemengist A Gebrie, Dejene A Tessema, Argaw Ambelu

**Affiliations:** 1 Department of Chemistry, College of Natural Sciences, Jimma University, Jimma, Ethiopia; 2 Department of Environmental Health Sciences, College of Public Health, Jimma University, Jimma, Ethiopia

**Keywords:** Blood lead level, Construction workers, Non-specific symptoms, Self-protection device

## Abstract

**Background:**

No study has been carried out to assess the blood lead levels of workers or the contribution of common workplace practices to lead exposure in Ethiopia. This study was carried out to assess the blood lead levels of female and male laborers in the construction sector in Jimma town, Ethiopia.

**Method:**

A cross-sectional study on the blood lead levels of 45 construction workers was carried out in the town of Jimma. The t-test, analysis of variance, the Kruskal-Wallis, Mann–Whitney and odds ratio tests were used to compare mean blood lead levels and to investigate the associations between specific job type, use of self-protection device, sex, service years and occurrence of non-specific symptoms with BLLs.

**Results:**

The mean blood lead level of the exposed group (40.03 ± 10.41 μg/dL) was found to be significantly greater than that of the unexposed group (29.81 ± 10.21 μg/dL), *p* = 0.05. Among the exposed group female workers were found to have higher mean blood lead level (42.04 ± 4.11 μg/dL) than their male colleagues (33.99 ± 3.28 μg/dL). Laborers who were regularly using self-protection devices were found to have significantly lower blood lead levels than those who were not using.

**Conclusion:**

The blood lead levels of construction workers in Jimma town are considerably high with a range of 20.46 – 70.46 μg/dL and the workers are in danger of imminent lead toxicity. More endangered are female construction workers who are bearers of the future children of the country and the issue requires urgent attention.

## Background

Lead is a cumulative and persistent toxic substance that poses a serious health risk to humans. In the general population, lead exposure arises from food, drinking water, occupation, hobbies or, to a lesser degree, atmospheric lead contamination [1]. Several investigators have also observed that lifestyles such as smoking cigarettes and alcohol contribute to lead uptake in humans [2-6]. Lead intoxication is dangerous to all people in all sectors but children are more vulnerable due to their lifestyle, rapid growth and still developing systems. Urban children in developing countries are known to be most at risk of lead intoxication and it was estimated in 1994 that over 80% of those between three and five years of age and 100% under two had average blood lead levels (BLLs) exceeding the threshold of 10 mg/dL set by the United States Centre for Disease Control (US-CDC) [3,5]. Children intoxicated with lead are more likely to have birth defects, mental retardation, or behavioural disorders or to die during the first year of childhood [7].

Once in the body, lead circulates in the blood; while most is excreted, some can remain in the tissues, organs and bones. From the skeleton as a primary storage site, lead gradually reenters the circulation through bone resorption [8]. In pregnant women, lead sequestered in the bone from past exposure or that accumulated in the body from recent exposure can be carried to the unborn baby by mobilization via the placenta. As a result, exposure to lead for women of childbearing age can have adverse effects on their offspring, should they become pregnant [6].

Exposure to lead is characterized by subtle, non-specific symptoms that frequently contribute to misdiagnosis of lead poisoning and the onset of symptoms depend on whether the intoxication was acute or chronic. Signs of chronic lead poisoning include weakness, excessive tiredness, irritability, constipation, anorexia, abdominal discomfort (colic), fine tremors and ‘wrist drop’. Overexposure to lead may also result in damage to the kidneys, anaemia, high blood pressure, impotence, infertility, and reduced sex drive in both sexes [9]. Although the WHO and CDC defined BLLs of 25 μg/dL as toxic in 1991, the scientific consensus is that there is no exposure level below which lead appears to be safe and action is being taken to reduce the international accepted levels of action [10,11]. Estimates published recently suggest that the theoretical minimum risk may occur at blood lead concentrations as low as 0 – 1 μg/dL [12]. Believing that some of the effects of lead, particularly changes in the levels of certain blood enzymes and in aspects of children’s neurobehavioral development, may occur at blood levels so low as to be essentially without a threshold, the US Environmental Protection Agency (EPA) and US Agency for Toxic Substances and Disease Registry (ATSDR), declined to specify a level of exposure not likely to lead to adverse effects for lead [9]. For lead, therefore, any exposure is of potential concern.

Lead poisoning has been identified as an occupational hazard from ancient times. A wide variety of industrial populations such as: people who work in lead smelting and refining industries and those who deal with lead during the manufacturing process, construction workers and people who work in pottery and ceramic industries could all be exposed to high lead levels [13]. In Ethiopia the construction sector is currently employing a huge number of unskilled general labourers, both men and women. Due to the better pay that the construction sector offers, many women who were working as maids in private homes and those who were jobless are joining the sector. They are employed mostly as unskilled labourers and are involved in jobs such as: demolishing, manual scraping, concreting, plastering, painting, etc. Most of these laborers eat their lunch at the work place sitting at any corner in the site under dusty conditions. They have no access to wash their hands before they eat. As lead is most commonly absorbed into the body by inhalation, the workers can absorb lead dust produced when metal is cut or when lead paint is windblown during demolition of old houses that existed on the construction sites or, from the fumes resulting from manual spray painting of metallic doors [14]. Moreover, lead dust can settle on the hands and clothes of the workers when they handle lead containing activities. They can then ingest the lead that has settled on their hands and/or on their food when they eat, drink or smoke without washing their hands. So, really at issue is that they are not aware of the potential dangers and have not had Workplace Hazardous Materials Information System (WHMIS) training and even if they did, there is little beyond using face masks, which they could do to protect themselves and their families. As a result, the level of lead in construction workers in general can be greater than that of the general population and the level in females may also be greater than the maximum tolerable limit in women of child bearing age.

In Ethiopia, no investigation has been carried out on the occupational lead exposure of workers except the cross-sectional study carried out in Addis Ababa on workers in lead acid battery repair units of transport service enterprises, and the study carried out in Jimma on the BLL of auto-garage workers [15,2]. In addition to this, there are no statistical data that show the number of workers at risk due to lead exposure. Therefore, the main objective of this research was to determine the BLLs of unskilled male and female labourers working in the construction sector in Jimma town and compare their relative workplace exposure to lead. However, since the BLL of the general population is not monitored in Ethiopia, reference ranges of BLLs that can be used by researchers or physicians to determine whether an individual has been exposed to higher levels than are found in the general population are not established. As a result individuals who were not working in the construction sector were included in this study to find out whether the BLLs and associated non-specific symptoms in the construction workers were significantly different from those of the general population.

## Materials and methods

### Study area and sample selection

Jimma is a small town in South West Ethiopia. There are no major industrial sources of lead emission in the town. There are however, several small scale metal workshops, auto-garages and lead acid battery repair units of transport service enterprises and nearly all are located in the center of the town. The central bus station of the town is also found in the town center and emissions from car fuel exhaust and from other industrial activities are high in this part of the town. Residents of this part of the town are expected to be exposed to ‘high’ amount of atmospheric lead relative to residents living in areas ‘far’ from the center.

The current growth in the construction sector in the town has attracted many jobless young male and female residents of the town or from the surrounding rural areas. Three construction sites with relatively large number of workers (50 – 75) were purposively selected for the current study. About two thirds of the laborers in the selected construction sites were females. With the permission of the respective site managers, the workers were approached and requested to participate in the study on a voluntary basis after the purpose of the study was clearly explained to them. Finally, 35 female and 10 male workers (occupationally exposed) were found to fulfill the inclusion criteria (a duration of at least 6 months on the job and, residential place ‘far’ , at least 3 kms, from the town center) and all agreed and participated in the study voluntarily. Oral and written consent was obtained from each participant. Thirty female and fifteen male boarding students of Jimma University who had apparently no history of lead exposure, were non-smokers, non-alcoholics and supposed to be unexposed, were selected from among those who stayed in the University for at least 6 months and asked to participate in the study. Five of the female students and four of the male refused to donate blood samples while twenty five females and eleven males expressed their willingness and donated blood samples. This group served as the control group. The dormitories of the selected students were located about 5 kms North-East of the town center. The study was conducted after obtaining ethical approval from the Ethical and Research Committee of the College of Public Health and Medical Sciences at Jimma University.

### Reagents and chemicals

Analytical standard solutions of lead (Spectro ECON) were prepared by serially diluting a 1000 mg/L stock calibration standard solution. Reagents used included trichloroacetic acid (5%; TCA) and perchloric acid (extra pure, Riedel-de Haen, Germany), K_2_EDTA and ammonium pyrollidine dithiocarbamates (APDC; Merck (Darmstadt), calcium chloride and methyl iso-butyl ketone (MIBK; Sigma Chemical Co).

### Blood sample collection and analysis

A venous blood sample (about 5 mL) was collected from each study unit by qualified medical laboratory professionals into a labeled heparinized blood collection vacutainer tubes containing 7.2 mg of K_2_EDTA. Separate sterilized needle and glove was used for each individual. The samples were homogenized by hand shaking and 2 mL from each sample was transferred into separate pyrex test tubes. To this, 4.0 mL of 5% TCA was added to make the Blood:TCA volume ratio 2:1. To this mixture, 1.3 mL of 2 M perchloric acid, 0.8 mL of APDC- surfactant solution, 50 μL 1.5 M CaCl_2_, and 2.0 mL water saturated MIBK were added successively and shaken for about 20 seconds. The resulting mixtures were centrifuged for 10 minutes at 2000 rpm using PLC-02 Gemmy, Taiwan, centrifuge. The Pb-APDC solution was analyzed in MIBK within 2 hours of extraction. All used needles and gloves were packed in appropriately labelled disposable bags and disposed into the Jimma University Specialized Hospital waste disposal unit.

The concentration of lead in the blood samples was determined by Flame Atomic Absorption Spectrometer (NovAA 300) at 283.3 nm after optimizing the various analytical parameters. Blood from each study unit was measured in triplicate and averages of triplicate measurements were taken for each experimental unit participant’s sample. Instrument drift was controlled by running standards after the successive analysis of 10 samples. Quantification of lead in blood was done with the use of a standard curve (0–2 ppm) prepared from a commercial lead standard. For quality assurance, due to unavailability of Standard Reference Material (SRM) in our laboratory, validation of the analytical protocol was done by the standard addition method of the metal. Percentage recoveries determined from blood samples fortified with 10 μg of lead per 4.0 mL of blood sample averaged 94.6%. However, no correction for recoveries was performed on the data. The limit of detection for lead was determined from the concentration equivalent of three times the standard deviation of 7 determinations at 0.05 ppm lead. Uniformity of variance was tested by carrying out 7 additional determinations at 0.1 ppm lead solution. The limit of detection determined from the pooled standard deviation was found to be 0.054 mg/L.

### Data collection

In addition to blood lead concentration analysis, standardized questionnaires, designed to yield the required information from each participant, consisting of 35 questions for the exposed and 29 for the unexposed were completed during face-to-face interviews privately. Both groups answered the same 29 questions regarding demographic data, socioeconomic status, habits, perceived health, and non-specific symptoms related to elevated BLL. The remaining 6 questions were specific to the exposed group. The questionnaires were prepared in English but translated into the local language for those who did not understand English.

### Data analysis

Data analysis was carried out using SPSS version 16.0. Statistical analysis of BLLs and other data obtained through questionnaire was carried out by categorizing the exposed and/or unexposed groups by sex, specific job type, occurrence of non-specific symptoms, alcohol consumption habit and service year. Descriptive statistics was used to cross tabulate factors by study groups. The parametric independent *t*-test, was used for the comparison of mean BLLs of exposed and unexposed cohorts. Comparisons of skewed categorical data were performed by using the non-parametric Kruskal Wallis and Mann–Whitney *U*-tests. The Fisher’s Exact Ratio test was used to evaluate the occurrence of non-specific symptoms among the individuals in the exposed group relative to those of unexposed group. The chi-square was used to test the dependence of BLL on service year.

## Results and discussion

### Characteristics of study units

**Figure 1:**
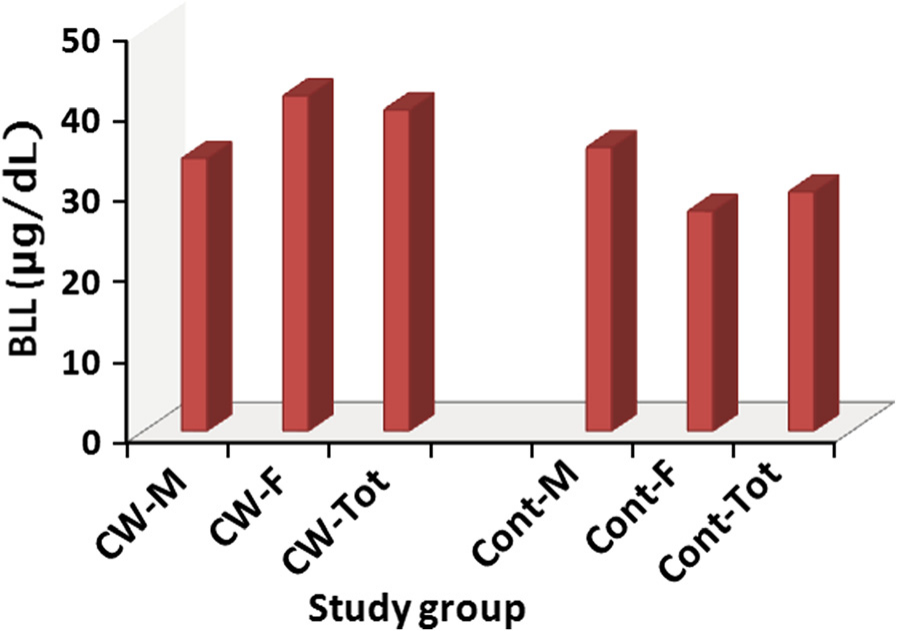
**Mean BLLs of the construction workers and non-construction workers.** M (male), F (female), CW (construction workers), Cont (controls), Tot (total).

A total of 45 occupationally exposed (35 females and 10 males) construction workers and 36 unexposed (25 females and 11 males) individuals participated in the study. The ages of the occupationally exposed group ranged between 18 and 45 years while that of the unexposed group ranged between 18 and 30 years. According to the Mann–Whitney *U*-test (two tailed, *p* = 0.05) the ages of the two groups did not differ significantly. All the construction workers stay at work for 8 – 10 hours per day for six days in a week. Results of the blood lead concentration analysis are given in Figure 1. According to the *t*-test, the mean BLL of the exposed cohort, 40.03 μg/dL (95% CI: 36.9 – 43.16, median: 38.89 μg/dL; range: 20.46 – 70.46 μg/dL) was significantly greater than that of the unexposed cohort, 29.82 μg/dL (95% CI: 26.36 – 33.28, median: 30.26 μg/dL; range: 12.43 – 46.65 μg/dL) (*p* < 0.05).

The mean BLLs of the females and males in each of the two cohorts was also calculated and, that of female workers in the exposed group (42.04 ± 4.11 μg/dL, range: 21.64 – 58.76 μg/dL), was found to be higher than that of their male colleagues (33.99 ± 3.28 μg/dL, range: 20.46 – 38.12 μg/dL), female members of the unexposed group (27.28 ± 2.76 μg/dL, range: 12.43 – 46.65 μg/dL), and male members of the unexposed group (35.35 ± 4.48 μg/dL, range: 12.78 – 45.84 μg/dL). According to the Kruskal-Wallis test the BLLs of the four groups were found to be significantly different at *p* = 0.05. The Mann–Whitney *U*-test performed between pairs of groups has also shown that the BLL of the female workers in the exposed group was significantly greater than that of each of the other groups (*p* < 0.05). On the other hand, in the unexposed cohort the mean BLL of males was significantly higher than that of the females (*p* < 0.05).

The BLLs in the exposed and unexposed cohorts were categorized into different arbitrary ranges and the number of individuals with BLLs in each of the ranges in the two cohorts was calculated and the results are given in Table 1. The BLLs of all individuals in the exposed workers were greater than 20.46 μg/dL, while 22% of the unexposed group had BLLs of between 10 and 20 μg/dL. The remaining 77.78% of the unexposed group had BLLs in the range 20–50 μg/dL and none of them had a BLL above 50 μg/dL. On the other hand, 26.67% of the exposed group had BLL above 50 μg/dL. One person among the exposed group had higher BLL, 70.46 μg/dL, and specific reasons for the relatively higher BLL of the person were not clear.

**Table 1:** Number of the exposed and unexposed individuals with BLLs in different concentration ranges

	**№ and% of individuals in the exposed group**		**№ and% of individuals in the unexposed group**	
**BLL range (μg/dL)**	**n (n**_**M**_**, n**_**F**_**)**	**% T (% M, % F)**	**n (n**_**M**_**, n**_**F**_**)**	**% T (% M, % F)**
10 – 20	0	0	8 (1, 7)	22 (2.8, 19)
20 – 35	13 (7, 6)	28.89 (15.6, 13)*	15 (4, 11)	41.67 (11, 30.6)
35 – 40	12 (2, 10)	26.7 (4.5, 22)	6 (1, 5)	16.67 (2.8, 13.9)
40 – 45	7 (0, 7)	15.6 (0, 15.6)	5 (4, 1)	13.89 (11, 2.8)
45 – 50	5 (0, 5)	11(0, 11)	2 (1, 1)	5.56 (2.8, 2.8)
50 – 55	4 (0, 4)	8.9 (0, 8.9)	0	0
55 – 60	7 (0, 7)	15.6 (0, 15.6)	0	0
Above 60	1 (1, 0)	2 (2, 0)	0	0

n = total number of individuals, n_M_ = number of males, and n_F_ = number of females.

% T = % of both males and females, % M = % of males, % F = % of females.

*Values in bracket indicate the percent share of males and females respectively.

Among members of the exposed group, all males had BLLs in the range of 20 and 40 μg/dL and all the higher BLLs were recorded for female members except one, 70.46 μg/dL, that corresponds to male member of the exposed group. In the unexposed group the lower BLL ranges are dominated by females while the higher ranges, specifically 40 – 45 μg/dL, was dominated by males.

### BLL and age relationship

**Figure 2:**
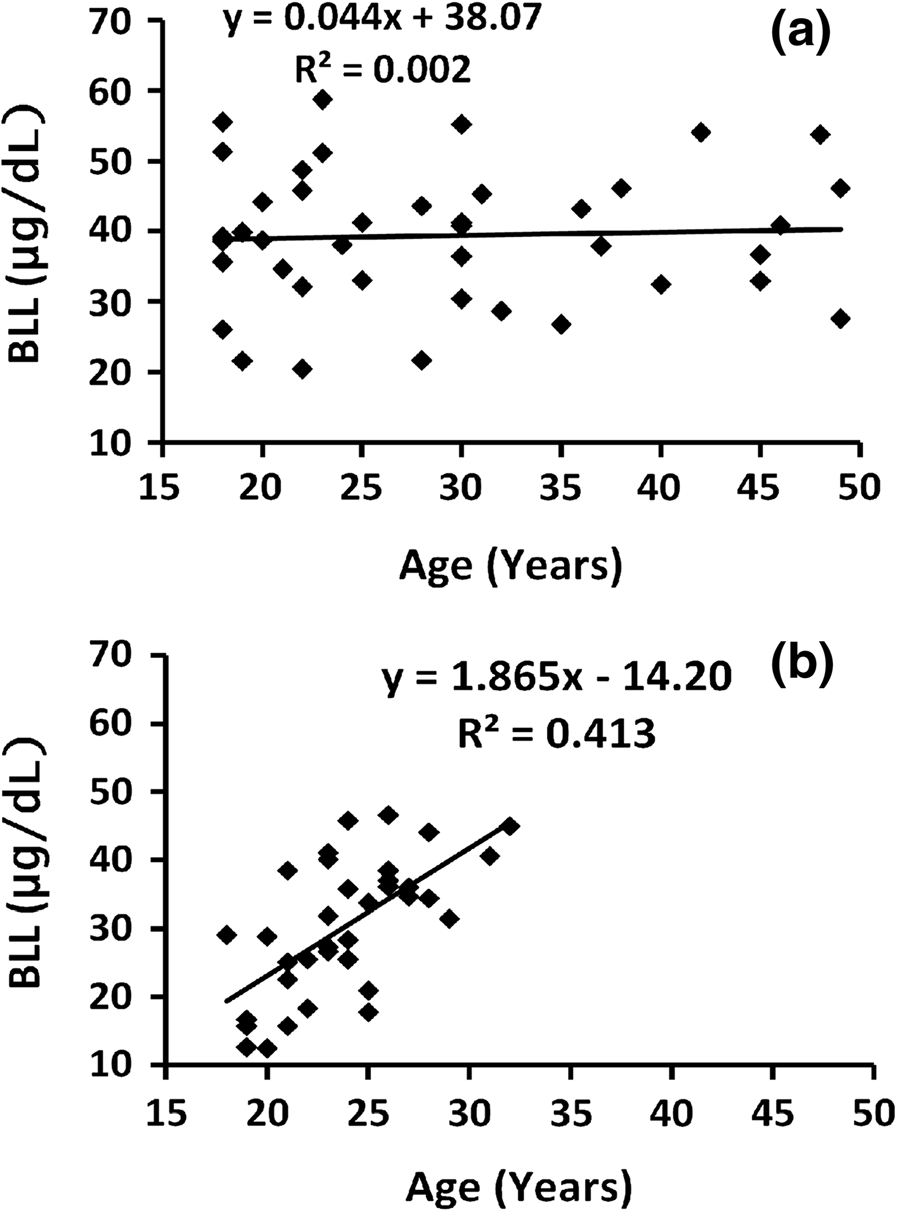
Age versus BLL of construction workers (a) and non-construction workers (b).

The regression of age on BLL was calculated for both the exposed and unexposed cohorts and the results obtained are depicted in Figure 2. As can be seen clearly from the figure, age and BLL are not correlated in the exposed group while a positive correlation with R^2^ value of 0.413 was obtained for the unexposed group.

The mean BLLs of the workers in different age categories were calculated and the results are shown in Table 2. As it is indicated in the table, the majority of the workers from both sexes were between 18 – 25 years and the mean BLL of this age group was observed to be lower than that of the higher age groups. The difference however, was not statistically significant. The majority of female members of the exposed group (about 75%) were in the most fertile life stage (18 – 35 years). According to the Mann–Whitney *U*-test the observed difference in the mean BLLs of these members of female workers (41 μg/dL) and that of the older female members (45 μg/dL) were not statistically significant at the 95% confidence level.

**Table 2:** Mean BLL and number of the exposed individuals in different age group

	**Number (%) of workers**			**Mean BLL (Mean ± SD) μg/dL)**		
**Age (Years)**	**Females**	**Males**	**Total**	**Males**	**Females**	**Total**
18 – 25	17 (48.6)^a^	6 (60)^b^	23 (51.1)^c^	29.3 ± 7.1	39.7 ± 6.7	36.3 ± 7.3
26 – 35	10 (28.6)^a^	3 (30)^b^	13 (28.9)^c^	32.2 ± 6.4	46.8 ± 8.5	44.4 ± 9.8
36 – 45	4 (11.4)^a^	1 (10)^b^	5 (11.1)^c^	33*	47.7 ± 7.5	44.8 ± 9.2
46 – 49	4 (11.4)^a^	-	4 (8.9)^c^	-	46.2 ± 3.6	46.2 ± 3.6

^a^percent from female workers, ^b^percent from male workers, ^c^percent from total workers. *only one person.

### BLL and specific job type

The mean BLLs of the exposed workers involved in different job categories were calculated and the results are given in Table 3. The workers involved in painting had a higher mean BLL (44.55 ± 9.47 μg/dL) than those of the concrete mixers and/or stone carriers (36.65 ± 8.48 μg/dL), and plasterers (39.74 ± 11.79 μg/dL). According to the Kruskal-Wallis test however, the differences were not statistically significant (*p* > 0.05).

**Table 3:** The mean, maximum and minimum BLLs of the construction workers involved in different job categories

**Labor of division**	**n**	**BLL (μg/dL)**		
		**Mean ± SD**	**Minimum**	**Maximum**
Painters	13	44.55 ± 9.47	26.82	58.76
Stone carriers/concrete mixers	15	36.65 ± 8.48	20.46	48.76
Plasterers	17	39.76 ± 11.79	21.64	70.46

### Use of SPD and alcohol drinking habit

**Figure 3:**
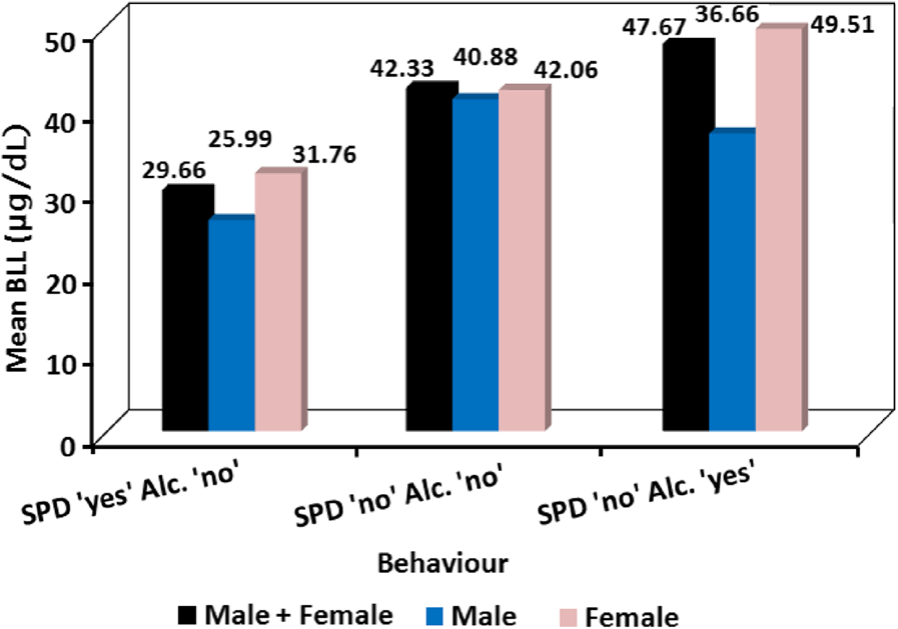
**BLLs of groups with different behaviors of using SPD and drinking alcohol.** SPD ‘yes’ Alc. ‘no’ = workers using SPD and do not drink alcohol regularly; SPD ‘no’ Alc. ‘no’ = workers who do not use SPD nor drink alcohol regularly; SPD ‘no’ Alc. ‘yes’ = workers who do not use SPD and drink alcohol regularly.

The number of workers who reported to consume alcohol was only 20% of each of the male and female workers and all of them expressed that they consume alcohol at least four days in a week. The correlation between BLL and the use of SPD and/or alcohol consumption was evaluated from the data collected through questionnaire and the results are depicted in Figure 3.

As can be seen from the bar graph the mean BLL of members of the exposed group who were using SPD while at work and who were not alcohol consumers, was 29.66 ± 6.15 μg/dL (males 25.99 ± 5.95 μg/dL, n = 4, females 31.76 ± 6.51 μg/dL, n = 7). On the other hand, the mean BLL of the members who were not using SPD while at work and were not alcohol consumers was 41.33 ± 9.36 μg/dL (males 40.59 ± 17.13 μg/dL, n = 5; females 42.74 ± 7.07 μg/dL, n = 21); and that of members who were not using SPD and were alcohol consumers was 47.67 ± 8.26 μg/dL (males 36.66 μg/dL (n = 1), females 49.51 ± 7.32 μg/dL, n = 6). According to the Kruskal-Wallis test, the observed difference between the BLLs of the three groups was significant (*p* < 0.05).

### BLL and service year

**Figure 4:**
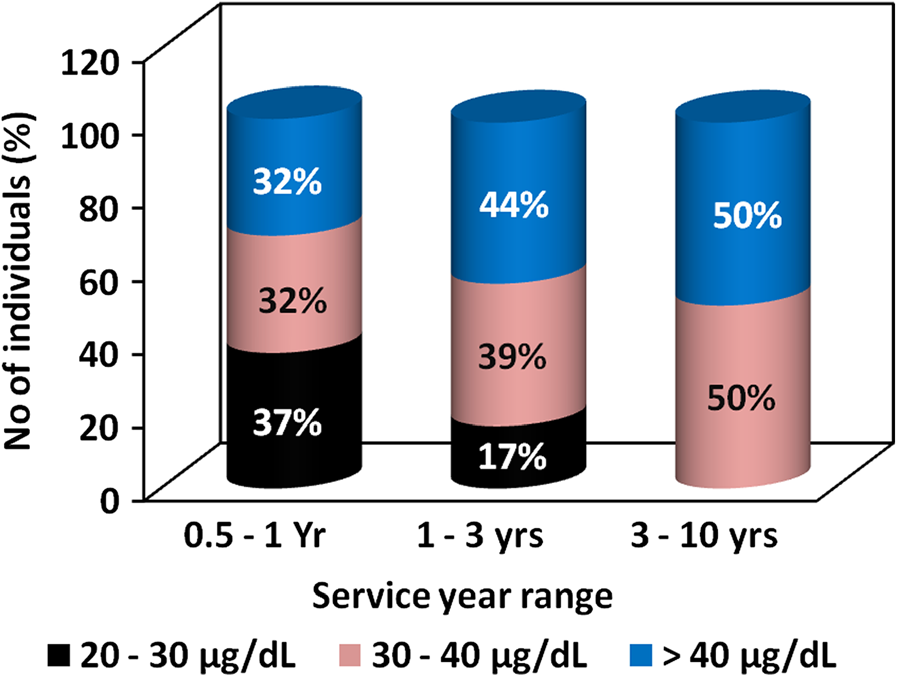
Proportion of individuals with BLLs between 20–30 μg/dL, 30–40 μg/dL and above 40 μg/dL among the construction workers with service years between 0.5 – 1 year, 1 – 3 years and 3 – 10 years.

The proportion of individuals with BLLs between 20 to 30, 30 to 40 and or above 40 μg/dL among the construction workers with service years between 0.5 – 1, 1 – 3 and 3 – 10 years are depicted in Figure 4. The figure clearly shows a steady increase in the proportion of individuals with higher BLLs with an increase in service years. Among the workers in the 0.5 – 1 service year group, the relative number of individuals with BLLs between 20 – 30 μg/dL (37%) was greater than that of individuals with BLLs ranging 30 – 40 (32%) and above 40 μg/dL (31%).

According to the chi-square test the observed difference was not, however, significant at *p* = 0.01. On the contrary, the relative number of individuals with BLLs ranging 20 – 30 μg/dL in the 1 – 3 service year group was lower than that of individuals with BLLs between 30 – 40 or greater than 40 μg/dL. Among the workers in the 3 – 10 service year group no worker had a BLL in the 20 – 30 μg/dL range. In the 1 – 3 service year group 39 and 44% of individuals were found to have BLLs between 30 – 40 μg/dL and greater than 40 μg/dL respectively, while among those in the 3 – 10 service year group, those with BLLs between 30 – 40 and greater than 40 μg/dL were 50% each. The chi-square test has revealed that the difference in the number of individuals with BLLs between 20 – 30, 30 – 40 and greater than 40 μg/dL among the workers in 1 – 3 or 3 – 10 service year categories was significant (*p* = 0.01). The difference in the number of individuals with BLLs between 30 – 40 and greater than 40 μg/dL in both service year groups, however, was not significant (*p* = 0.01). With an increase in service year, the proportion of individuals with lower BLL was found to decrease while that of individuals with higher BLLs was increasing. This clearly shows the direct relationship between BLL of the construction workers and service year.

### Lead toxicity symptoms

The Odds ratio of the reported non-specific symptoms in the construction workers in relation to the controls group was calculated and the results obtained are shown in Table 4. The results indicate that among the reported non-specific symptoms, the occurrence of memory impairment, headaches, wrist drop, nausea and abdominal discomfort in the construction workers are significantly greater than in the control group.

**Table 4:** Reported symptoms among the exposed (n = 45) and the unexposed (n = 36) groups and the ratios of their odds

	**№ and% of ‘yes’ response for symptom**				
**Symptom**	**Exposed**	**Unexposed**	**OR**	**95% CI**	**p-value**
Depression	3 (6.67)	1 (2.78)	2.5	0.25 - 25	0.39
Memory impairment	9 (20)	2 (5.56)	4.25*	0.86 - 21	0.05
Sleep disturbance	3 (6.67)	4 (11.11)	0.57	0.12 – 2.7	0.79
Concentration difficulty	6 (13.33)	2 (5.56)	2.62	0.49 – 14	0.22
Headaches	23 (51.11)	2 (5.56)	17.77*	3.8 - 83	0.00
Wrist drop	9 (20)	1 (2.78)	8.75*	1.1 – 73	0.02
Lack of appetite	3 (6.67)	1 (2.78)	2.5	0.25 - 25	0.39
Nausea	8 (17.77)	1 (2.78)	7.57*	0.9 - 64	0.03
Constipation	3 (6.67)	1 (2.78)	2.5	0.25 - 25	0.39
Abdominal discomfort	25 (55.56)	12 (33.33)	2.5*	1.0 – 6.2	0.04

OR = Odds Ratio.

*Significant relative risk of occurrence in the construction workers at p < 0.05.

## Discussion

Since 1980, when the WHO specified a BLL of 40 μg/dL in the general population and 30 μg/dL in women of childbearing age, the so called “safe BLL” in humans has undergone a number of revisions with the advancement of knowledge on the toxicity of lead at lower concentrations. Although no threshold has been determined for the harmful effects of lead, the Centers for Disease Control and Prevention (CDC) considers BLL ≥10 μg/dL to be of health concern for children, and BLLs ≥25 μg/dL for adults [16]. Moreover, a UNEP-UNICEF document advises the beginning of treatment within 48 hours for BLL exceeding 45 μg/dL and a BLL more than 70 μg/dL as presenting a medical emergency [17]. However, a number of recent studies demonstrate that lead inflicts significant damage even at BLLs below 5 μg/dL and recommends that level be kept below 10 μg/dL and, pregnant women or women considering pregnancy should not have a BLL above 5 μg/dL [18-20]. The results of this study have revealed that the BLL of unskilled construction workers ranges between 20.46 μg/dL and 70.46 μg/dL and that of non-construction workers between 12.43 μg/dL and 46.65 μg/dL. The difference between the BLLs of the two groups was significant. The BLL results in this study indicate that, both the general population and the construction workers are exposed to lead but the exposure of the construction workers is significantly higher than that of the general population.

Sixteen (35%) of the construction workers and 2 (5.7%) of the unexposed group had BLLs exceeding 45 μg/dL. According to the suggestion given in the UNEP-UNICEF document, these were people who should have begun treatment within 48 hours [17]. The BLL of one person in the exposed group was at the border of the limit considered to present a medical emergency. Other studies on the occupational exposure of different groups have reported similar ranges of BLLs with that of this study. For example, Fatoki and Ayoade assessed the level of lead in blood samples of occcupationally and non-occupationally exposed donors in Ile-Ife, Nigeria, and they found a BLL range of 28.75 ± 17.3 μg/dL – 35.5 ± 15 μg/dL in the occupationally exposed cohorts [21]. A cross-sectional study conducted to determine the status of environmental and occupational lead exposure in Nairobi, Kenya, reported a wider BLL range for the occupationally exposed group, 5.8 to 65 μg/dL, with a mean value of 22.6 ± 13.4 μg/dL [22]. Although the mean value in the Nairobi study is much lower, the BLL range of the group studied is similar to that of this specific study in Ethiopia. Aliasgharpour and Hagani determined the BLLs of a group of 31 male non-smoking industrial workers and found BLL ranging between 15.50 μg/dL and 59.99 μg/dL [23]. Rabin and co-workers reported that 63% of 381 registrants in the Massachusetts Occupational Lead Registry with BLLs of 40 μg/dL or higher are construction workers [24]. Their finding was slightly greater than that of this study in which the percent of construction workers with BLLs of 40 μg/dL and above were 53.34%. A study conducted in United Arab Emirates (UAE) on 100 industrial workers (exposed) and 100 non-industrial workers (non-exposed) to determine the effect of blood lead on the health of industrial workers reported a mean of BLL of 77.5 ± 42.8 μg/dL in the industrial workers and 19.8 ± 12.3 μg/dL in the non-industrial workers [25].

Following recommendations of the Council of State and Territorial Epidemiologists (CSTE) in 2009, the CDC/NIOSH Adult Blood Lead Epidemiology and Surveillance (ABLES) program changed their case definition of elevated BLL from greater than 25 μg/dL to greater than10 μg/dL based on evidence linking lower levels of lead in adults with decreased kidney function, cardiovascular disease and cognitive impairment [16]. The BLLs of the unexposed group in this study were all above 10 μg/dL, higher than the current CDC/NIOSH definition of elevated BLL, and 77.78% of them had BLL exceeding 20 μg/dL. A BLL exceeding 20 μg/dL is known to indicate a substantial exposure to lead. The findings of the current study are in accordance with the findings in our previous study [2], which showed a similar high BLL in the general population. This may be a clear indication of the fact that the general population is also exposed to lead from various other sources in the environment. In general, the community is exposed to lead from food, water, and air that may be present in hazardous concentrations. A similar report in which students and individuals working in an open environment with greater exposure to environmental pollution was given by Yakub and Iqbal [26].

The results of this study have also shown that, the mean BLL of the construction workers who were using SPD was significantly lower than that of the construction workers who were not using SPD while at work. Among the female construction workers, only 20% were SPD users while 60% of the male construction workers were using SPD while at work. The female construction workers who were not using SPD were also found to have significantly higher BLL than their female colleagues who were using SPD. These findings suggest that the use of SPD can significantly reduce the level of lead exposure by construction workers. Moreover, among the construction workers who were not using SPD, the BLL of those who were alcohol consumers was significantly higher than that of their colleagues who were not consuming alcohol. This might indicate the possible enhancement of lead accumulation in the blood by alcohol. Similar findings were reported by other researchers [2-6].

In Ethiopia only men were involved in construction related jobs some 10 to 15 years ago. Most uneducated jobless women were employed in private homes as maids. Currently however, due to the better pay that the construction sector offers, many of these women and those who had no job at all, are joining the construction sector and their number is increasing from time to time. All of these women are employed as unskilled labourers and are involved in all job types their male colleagues are involved in: demolishing, manual scraping, concreting, plastering and painting. Among the exposed group, in the current study, the BLL of females was found to be significantly greater than that of males. The findings of this study have also shown that the number of female construction workers who were using SPDs while at work was significantly lower than that of males. Statistical analysis has shown a positive correlation between BLL and not using SPDs.

In most Ethiopian families, due to cultural taboo, the routine house hold activities such as food preparation are undertaken by females only. Males are not expected to get into kitchen and prepare food, clean dish or do some other related activities. The females use clay pans for baking ‘injera’ , leavened bread prepared by fermentation of teff (*Eragrostis abyssinica Schrad*), wheat, barley, maize or sorghum, or from a mixture of these. They also use clay or metallic bowls for the preparation of ‘wot’ , a spiced sauce prepared from legumes, vegetables or meat depending on the income of the family. Therefore, women can be exposed to any lead originating from the materials they handle while carrying out these activities which are not handled by men. This could be a possible confounder in our study. Therefore, we cannot exclusively conclude that lack of using SPDs in the work place is the only reason for the higher BLLs of the female workers than those of males. Another possible reason for the higher BLL in females, reported in the literature, could be sex difference either in uptake or retention of lead. In a comparison between the relative retention of lead by men and premenopausal women, it has been found that, premenopausal women retain lead more avidly or release lead more slowly than do men and pointed out that this distinction is lost for postmenopausal women [8]. This conclusion fits that of our observation since all the women included in our study were premenopausal, according to the responses they gave during interview.

The mean BLL of the unexposed group was found to be positively correlated with age while that of the exposed group was not. This might be a relation that would discriminate between occupational and non-occupational lead exposure. Gradual exposure to environmental lead by the non-occupationally exposed group may cause gradual increase in BLL with age. In the exposed group, however, exposure to high level of lead in a relatively shorter period at the work place in addition to the exposure from other environmental sources might cause the workers to accumulate high amount of lead in a shorter duration of stay in the work. Thus the positive correlation between age and BLL in the non-exposed group unlike in that of the exposed group seems to be reasonable. Similar positive correlation between BLL and age by the referent group was reported in the literature [8].

The American Conference of Governmental Industrial Hygienists (ACGIH) advises women of child-bearing age that if their BLL is >10 μg/dL, they are at risk of delivering a child with a BLL >10 μg/dL, which is the level of concern in the pediatric CDC guidelines [27]. Although research findings have been inconsistent, the results of some recent investigations indicate that maternal BLL <10 μg/dL may be related to adverse reproductive outcomes, including preterm birth and spontaneous abortion [28,29]. The fact that all the female construction workers and non-construction workers who participated in our study had BLLs >10 μg/dL and are in their most fertile age shows that they are parts of these risk groups.

The construction workers were found to exhibit significantly higher levels of the non-specific symptoms which included: memory impairment, headaches, wrist drop, nausea, and abdominal discomfort relative to the non-construction workers. Our report on the variations of the non-specific symptoms between the two groups is entirely from what the two groups revealed in the questionnaires and interviews. Although this may be suggestive of the adverse effects of lead on the exposed individuals relative to the unexposed, a close medical investigation is required to affirm that the epidemiologic variations between the two groups are exclusively results of the difference in the BLLs of the groups.

Our analysis might have been affected by several potential limitations of our study. Since confounding factors were not identified and controlled results of the various categories of the workers may be biased. In the workplace workers are not stationed to a specific working area. They move from places to place to help each other. Therefore, the BLL data corresponding to specific job types of workers may be biased because of such unrestricted movement. The sample sizes of both the occupationally exposed and unexposed cohorts might not be representative of the construction workers and the general population in Ethiopia. Similarly the female and male compositions in the two groups might not be representative of the two sexes to draw sex based conclusions. We had no records of environmental lead exposure in the proximity of the construction workers because monitoring of lead in air was not enforced in the country in general. As a result, any observed difference in response to occupational and environmental lead exposure may, therefore, be attributed to a degree of exposure to lead or different metabolic responses to lead in the construction workers. The participants in the control group were selected from boarding university students who have come from various rural and urban areas in the country where the exogenous lead exposures are different. As a result, any difference in BLL might not be attributed to the current living environment of the group. Moreover, absence of some epidemiological symptoms in this group might not be exclusively attributed to lower BLL relative to the construction workers.

## Conclusions

This study has shown that unskilled construction workers are more likely exposed to lead than the general population. The BLLs determined are also high enough to call for an urgent measure. Infact more urgent is the case of females whose exposure is directly related with the health of their future babies. When we consider the literature reports on the association between: prenatal and postnatal blood lead concentrations with higher rates of total arrests and/or arrests for offenses involving violence, childhood lead exposure with region-specific reductions in adult gray matter volume, the inverse association of blood lead concentration in children with IQ, the issue goes beyond mother and child; extends to country level [30-32]. Mentally unhealthy and violent children would definitely harm the socio-economic development of the country and the peaceful life of the society.

The higher BLL determined in the unexposed group has also revealed that the general population including children is exposed to lead from different sources in the environment. The society in general, including most educated ones, is not aware about the dangers and routs of lead exposure and there is no BLL monitoring program in the country. As a result people do not protect themselves and their children from exposure to lead. Therefore, we call for a further large-scale screening and regular monitoring of occupationally exposed and unexposed workers to save the community from long term adverse effects of lead exposure.

## Competing interests

The authors declare that they have no competing interests.

## Authors’ contributions

HAG carried out the sampling, analytical work and statistical analysis. AA participated in the design of the study and the statistical analysis. DAT conceived the study, participated in its design, statistical analysis and coordination and, prepared the manuscript. All authors read and approved the final manuscript.
